# Risk Factors for Suicidal Behaviour in Individuals on Disability Pension Due to Common Mental Disorders - A Nationwide Register-Based Prospective Cohort Study in Sweden

**DOI:** 10.1371/journal.pone.0098497

**Published:** 2014-05-28

**Authors:** Syed Rahman, Kristina Alexanderson, Jussi Jokinen, Ellenor Mittendorfer-Rutz

**Affiliations:** 1 Department of Clinical Neuroscience, Division of Insurance Medicine, Karolinska Institutet, Stockholm, Sweden; 2 Department of Clinical Neuroscience, Division of Psychiatry, Karolinska Institutet, Karolinska University Hospital, Stockholm, Sweden; University of Vienna, Austria

## Abstract

**Background:**

Common mental disorders (CMD) have become one of the leading causes for disability pension (DP). Studies on predictors of adverse health outcome following DP are sparse. This study aimed to examine the association of different socio-demographic factors and health care consumption with subsequent suicidal behaviour among individuals on DP due to CMD.

**Method:**

This is a population-based prospective cohort study based on register data. All individuals aged 18–64 years, living in Sweden on 31-Dec-2004 who in 2005 were on DP due to CMD (N = 46 745) were followed regarding suicide attempt and suicide (2006–10). Univariate and multivariate hazard ratios (HR) and 95% confidence intervals (CI) for suicidal behaviour were estimated by Cox regression.

**Results:**

During the five-year follow-up, 1 046 (2.2%) and 210 (0.4%) individuals attempted and committed suicide, respectively. Multivariate analyses showed that young age (18–24 years) and low education predicted suicide attempt, while living alone was associated with both higher suicide attempt and suicide (range of HRs 1.23 to 1.68). Combined prescription of antidepressants with anxiolytics during 2005 and inpatient care due to mental diagnoses or suicide attempt (2001–05) were strongly associated with suicide attempt and suicide (range of HRs 1.3 to 4.9), while inpatient care due to somatic diagnoses and specialized outpatient care due to mental diagnoses during 2001–05 only predicted suicide attempt (HR 1.45; 95% CI: 1.3–1.7; HR 1.30; 95% CI: 1.1–1.7).

**Conclusions:**

Along with socio-demographic factors, it is very important to consider type of previous healthcare use and medication history when designing further research or intervention aiming at individuals on DP due to CMD. Further research is warranted to investigate both characteristics of disability pension due to CMD, like duration, diagnoses and grade as well as mechanisms to subsequent suicidal behavior, taking potential gender differences into consideration.

## Introduction

The prevalence of disability pension is high in several European countries [1]–[4]. The reasons for this high prevalence are not entirely disentangled [2]. Considerable increases in the incidence of disability pension in several European countries also contrast the overall improvement in general health. It has been discussed that such increases may be due to changes in the society, including various social factors and factors related to working life [5].

Common mental disorders (CMD) may at any given time affect up to 15% of the general population [6]. However, they are often underreported, underdiagnosed, and undertreated [7], particularly when comorbid with other conditions [8]. CMD, including depression, anxiety, and stress-related mental diagnoses, comprise one of the main disability pension diagnostic groups [2], [3], [5], [9]. Such disorders can be positively affected by treatment and rehabilitation efforts and are likely to worsen with inactivity [10]. In Sweden, the proportion of mental diagnoses among all disability pensions more than doubled in the last 11 years, from 25 to 56% from 2000 to 2011 [11]. Moreover, the incidence of disability pension young adults increased considerably in Sweden. In 1995, 1931 people below the age of 30 years were granted disability pension; in 2011 that number had increased three-fold to 6 547 [11].

Psychological autopsy studies suggest, that about 90% of suicide completers had a mental disorder, primarily common mental disorders like depression and anxiety [12]. Comorbidity of these disorders additionally increased the risk of suicidal behaviour [13]–[18]. Gender differences in suicidal behaviour are well known, though it can differ according to geographical location. In Europe, women usually dominate in suicide attempt, while more men are suicide completers [12], [19], [20]. Different studies also reported, that attempted suicide is more common among the younger individuals and suicide risk is highest among the elderly [12], [21], [22]. Low educational level, living in urban areas, and being single have repeatedly been shown to be risk factors for suicidal behaviour [12], [20]–[23].

Studies also show that suicide risk is higher in individuals on disability pension [24]–[26], but to date there is no research on risk factors for suicidal behavior in this group. Despite the fact that knowledge on risk factors of negative consequences of being on disability pension is of crucial clinical and public health interest, the scientific evidence particularly with regard to adverse mental health consequences to date is lacking [27]. According to the existing scientific knowledge on health outcomes after being granted disability pension, the risk of all-cause and cause specific mortality, including suicide was found to be increased, regardless of the disability pension diagnosis [28]–[31]. The few previous studies have reported an association of disability pension with suicide in the general population [21], [28] or in subgroups of young disability pensioners [25].

The associations between different socio-demographic factors and disability pension are well recognized [32]–[34]. Age, sex, country of birth, place of residence, social status, education, occupation, employment conditions, marital status, parental status, et cetera have repeatedly been reported as important factors when studying this area [32]–[34]. However, currently there is a lack of knowledge on associations of such socio-demographic factors as well as factors related to health care and medication, on the risk of suicidal behaviour in disability pensioners.

### Aim

This study aimed to explore whether different socio-demographic factors, health care use, and medication are associated with suicidal behaviour (suicide and suicide attempt) in individuals on disability pension due to CMD.

## Methods and Materials

### Design

This is a population-based prospective cohort study based on nationwide register data. The study group included all individuals aged 18–64 years who lived in Sweden on 31-Dec-2004 and were on DP (full- and part time) due to CMD during all of 2005 (n = 48 803). Individuals with schizophrenic spectrum and bipolar disorders (n = 1 656) diagnosed in in- and outpatient health care during 2001–05 and people on old-age pension (n = 402) in 2005 were excluded. The final cohort thus included 46 745 individuals. Main diagnoses, according to the International Classification of Disease version 10, were used for inclusion or exclusion of individuals into the study group.

Annual data, both for the four years before inclusion at 01-Jan-2005 (2001–04) and till the end of follow-up 31-Dec-2010 were obtained from the following five nationwide registers: 1) Longitudinal integration database for health insurance and labour market studies (LISA) held by Statistics Sweden: including socio-demographic information on sex, age, educational level, type of living area, country of birth, family status; 2) Three registers held by the National Board of Health and Welfare, namely; (i) National patient register (NPR) including information on date and main diagnosis of in- and outpatient care, (ii) Cause of death register (CDR) with data on date and cause of death, (iii) Prescribed drug register (PDR) with data on type of medication, date of dispensing, available from July 2005; and 3) Micro-data for analyses of social insurance (MiDAS) with information on date and diagnoses of disability pension from the Social Insurance Agency. Data from different registers were linked at individual level, using the unique (de-identified) personal identification number of all residents in Sweden.

### Diagnostic criteria

All diagnoses were based on the corresponding codes of the International Classification of Diseases (ICD) version 10 (WHO 2010). CMD included ‘depressive episode’ (F32), ‘recurrent depressive disorder’ (F33), ‘phobic anxiety disorder’ (F40), ‘other anxiety disorder’ (F41), ‘obsessive-compulsive disorder’ (F42), and ‘reaction to severe stress and adjustment disorder’ (F43) [35], [36]. Antidepressant and anxiolytic prescriptions were based on the respective code in the Anatomical Therapeutic Chemical (ATC) Classification System N06A and N05B, respectively (WHOCC 2013). The excluded bipolar or schizophrenic spectrum disorder were also categorised according to ICD 10 (F20-F29, F31).

### Disability pension

All residents in Sweden aged 19–64 years, whose work capacity is permanently reduced due to disease or injury, are eligible to receive disability pension from the Social Insurance Agency [11]. Disability pension can be granted for 25, 50, 75, or 100% of ordinary working hours. Since 2003, people aged 19–30 years can be granted temporary disability pension if the work capacity is reduced for at least one year and in order to complete upper-secondary education [11].

### Risk factors

All baseline socio-demographic characteristics were measured on 31-Dec-2004 (age, sex, education, country of birth, place of residence, and family status). Eventual missing values of covariates have been coded as a separate category. The baseline characteristics except for in- and outpatient care and medication, were categorised as in table 1. In- and outpatient care (2001–05) due to mental/somatic diagnoses as well as inpatient care (2001–05) due to suicide attempt were dichotomised as ‘yes’ and ‘no’. Prescribed medication dispensed at least once in 2005 was categorised into ‘no medication’, ‘antidepressant only’, ‘anxiolytic only’, or ‘both prescribed’.

**Table 1:** Descriptive statistics of all 46 745 women and men, aged 18–64 years, living in Sweden on 31-Dec-2004, and on disability pension in 2005 due to common mental disorders.

Characteristics	Women		Men		All	
	N	%	N	%	N	%
**Study population**	31 003	66.3	15 742	33.7	46 745	100
**Age (years) in 2005**						
18–24	544	1.8	305	1.9	849	1.8
25–34	2 197	7.1	1 188	7.5	3 385	7.2
35–44	6 541	21.1	3 268	20.8	9 809	20.9
45–54	9 662	31.2	4 965	31.5	14 627	31.3
55–64	12 059	38.9	6 016	38.2	18 075	38.7
**Educational level (years)**						
Compulsory (0–9)	7 354	23.7	4 585	29.1	11 939	25.6
High school (10–12)	14 481	46.7	7 506	47.7	21 987	47.0
University (13 or >)	8 854	28.6	3 457	22.0	12 311	26.3
Unknown	314	1.0	194	1.2	508	1.1
**Type of living area** 1						
Big cities	12 536	40.4	6 958	44.2	19 494	41.7
Medium-sized cities	10 243	33.0	4 832	30.7	15 075	32.3
Small cities/villages	8 224	26.5	3 952	25.1	12 176	26.0
**Country of birth**						
Sweden	24 607	79.4	11 010	69.9	35 617	76.2
Other Nordic countries	1 519	4.9	615	3.9	2 134	4.6
EU 25 (except Nordic countries)	1 122	3.6	642	4.1	1 764	3.8
Rest of the world	3 755	12.1	3 475	22.1	7 230	15.5
**Family status**						
Marr/cohabit. with no children at home	7 136	23.0	2 868	18.2	10 004	21.4
Marr/cohabit. with children at home	7 162	23.1	3 674	23.3	10 836	23.2
Single^2^ no children at home	11 095	35.8	8 484	53.9	19 579	41.9
Single^2^ with children at home	5 610	18.1	716	4.5	6 326	13.5
**Diagnosis-specific specialized outpatient care in 2001**–**05**						
Somatic diagnosis	23 949	77.2	10 682	67.9	34 631	74.1
Mental diagnosis	8 333	26.9	4 757	30.2	13 090	28.0
**Diagnosis-specific inpatient care in 2001**–**05**						
Somatic diagnosis	10 664	34.4	4 972	31.6	15 636	33.4
Mental diagnosis	3 060	9.9	2 016	12.8	5 076	10.9
Suicide attempt	1 026	3.3	399	2.5	1 425	3.0
**Prescribed medication dispensed in 2005 (once or more)**						
Antidepressants only	9 608	31.0	3 851	24.5	13 459	28.8
Anxiolytics only	2 171	7.0	1 265	8.0	3 436	7.4
Both prescribed	5 049	16.3	2 222	14.1	7 271	15.6

1 Type of living area: Big cities: Stockholm, Gothenburg and Malmo; Medium-sized cities: cities with more than 90 000 inhabitants within 30 km distance from the centre of the city; small cities/villages;

2 Single means living without partner and includes divorces, separated or widowed

### Measurement of outcome

The outcome measure was defined as suicidal behaviour (suicide attempt or suicide) (ICD 10: X60-84 and Y10-34) during 2006–10. Suicide attempts and suicides are often underreported or reported as "undetermined" causes [37], [38]. Therefore, determined (X60–84) and “undetermined” (Y10–34) suicidal behaviour were combined in order to compensate for regional and temporal variation in ascertainment methods and to limit underreporting [39]. A sensitivity analysis was carried out to ensure that the estimates for determined and undermined suicidal behaviour were similar. The combined outcome measures are hereafter called suicide attempt and suicide.

### Statistical analysis

Individuals were followed until the event (suicide attempt, suicide), emigration, death (due to other causes in the analyses related to suicide as outcome), or end of follow up (31-Dec-2010), whichever occurred first. Univariate hazard ratios (HR) and 95% confidence intervals (CI) for the various predictors (socio-demographics, health care, and medication) with regard to the outcome were estimated by Cox proportional hazard regression models after testing that the proportionate hazard assumption was met. Multivariate models were built by stepwise adjusting and including only significant predictors and significant confounders. For test of interaction with sex, the partial likelihood ratio test was used. The proportion of suicide attempt during follow-up was estimated by life tables and plotted in 1- survival curves stratified by sex and separately according to previous suicide attempt and prescribed medication. Sensitivity analyses were carried out by calculating estimates (hazard ratios) and confidence intervals for determined and undetermined suicide attempt and suicide completion separately. In addition estimates (hazard ratios) and confidence intervals were also calculated after combining them. After assuring that these estimates were comparable, the combined variable was introduced into the model.

### Ethical statement

The study population was based on linkage of several public national registers. Ethical vetting is always required when using register data in Sweden. The ethical vetting is performed by regional ethical review boards and the risk appraisal associated with the Law on Public Disclosure and Secrecy is done by data owners. The ethical review boards can however waive the requirement to consult the data subjects (or in case of minors/children the next of kin, careers or guardians) directly to obtain their informed consent, and will often do so if the research is supported by the ethical review board and the data has already been collected in some other context. This means that for this specific study no written informed consent was given by participants (or next of kin/caregiver in the case of children) for their clinical records to be used. Patient records/information was anonymized and de-identified prior to analysis by the authority, Statistics Sweden, which was responsible for data linkage. Researchers received de-identified data.

This study was approved by the Regional Ethical Review Board of Karolinska Institutet, Stockholm, Sweden. (Dnr 2007/762–31, 2009/23–32, 2009/1917–32).

## Results

In total in Sweden, there were 46 745 individuals on disability pension due to CMD during 2005. Out of these (data not shown in table), 17 181 (36.8%) were on disability pension due to depressive episode, 11 022 (23.6%) had a diagnosis of reaction to severe stress and adjustment disorder, 9 799 (21.0%) due to other anxiety disorders, 4 950 (10.6%) due to recurrent depressive disorder, 2 783 (5.9%) received disability pension due to phobic anxiety disorder and 1 010 (2.2%) because of obsessive-compulsive disorder.

The majority (66.3%) of those on disability pension due to CMD in 2005 were women (table 1). Of the study population, 38.7% were aged 55–64 years, nearly half (47%) had been to high school, most lived in big or medium sized cities (74%), and 3 out of 4 were born in Sweden. Almost half of them (41.9%) were living without a partner and without children living at home. Furthermore, in the five years preceding start of follow up (2001–05), only 28% had had specialized outpatient care and 11% inpatient care due to mental diagnoses. Around 33% were hospitalized due to somatic diagnoses. Three percent of the cohort had been treated for attempted suicide (2001–05). While near one third of the disability pensioners were prescribed only antidepressants during the exposure year (2005), 16% were prescribed both antidepressants and anxiolytics (table 1).

In the cohort, 1 046 (2.2%) individuals attempted and 210 (0.4%) committed suicide during the five-year follow up (2006–10) (table 2). Women were more likely than men to attempt suicide (women: 2.4%, men: 2.0%) while a higher proportion of men completed suicide (women: 0.3%, men: 0.7%). Mean follow-up time for suicide attempt and suicide was around 5 years (4.85 (SD 0.70) and 4.91 (SD 0.52), respectively.

**Table 2:** Univariate hazard ratios for suicide attempt and suicide (2006–10), 46 745 women and men, aged 18–64 years and living in Sweden on 31-Dec-2004, and on disability pension in 2005 due to common mental disorders.

Characteristics	Suicide attempt			Suicide		
	n	%	Hazard ratio (95% CI)	n	%	Hazard ratio (95% CI)
***Socio-demographic factors***						
**Sex**						
Male	310	2	1	108	0.7	1
Female	736	2.4	1.2 (1.05–1.37)	102	0.3	0.47 (0.36–0.62)
**Age (years) in 2005**						
18–24	61	7.2	7.58 (5.67–10.15)	9	1.1	3.21 (1.59–6.47)
25–34	161	4.8	4.93 (3.98–6.10)	28	0.8	2.50 (1.60–3.92)
35–44	342	3.5	3.59 (2.99–4.31)	40	0.4	1.23 (0.83–1.84)
45–54	306	2.1	2.15 (1.79–2.59)	74	0.5	1.54 (1.09–2.17)
55–64	176	1.0	1	59	0.3	1
**Educational level (years)**						
Compulsory (0–9)	347	2.9	2.39 (1.98–2.89)	63	0.5	1.31 (0.90–1.90)
High school (10–12)	540	2.5	2.01 (1.68–2.40)	95	0.4	1.07 (0.76–1.51)
University (13 or >)	152	1.2	1	50	0.4	1
Unknown	7	1.4	1.13 (0.53–2.41)	2	0.4	0.98 (0.24–4.04)
**Type of living area** 1						
Big cities	407	2.1	1	89	0.5	1
Medium-sized cities	347	2.3	1.10 (0.95–1.27)	65	0.4	0.94 (0.68–1.30)
Small cities/villages	292	2.4	1.15 (0.99–1.33)	56	0.5	1.01 (0.72–1–40)
**Country of birth**						
Sweden	847	2.4	1	173	0.5	1
Other Nordic countries	73	3.4	1.47 (1.16–1.86)	10	0.5	0.98 (0.52–1.86)
EU 25 (except Nordic countries)	34	1.9	0.82 (0.58–1.15)	4	0.2	0.47 (0.18–1.27)
Rest of the world	92	1.3	0.53 (0.43–0.66)	232	0.3	0.66 (0.42–1.01)
**Family type**						
Marr/cohabit. with no children at home	88	0.9	0.48 (0.37–0.61)	29	0.3	1.06 (0.63–1.76)
Marr/cohabit. with children at home	201	1.9	1	30	0.3	1
Single^2^ no children at home	558	2.8	1.56 (1.33–1.83)	125	0.6	2.35 (1.58–3.50)
Single^2^ with children at home	199	3.1	1.71 (1.40–2.08)	25	0.4	1.43 (0.84–2.43)
***Health care factors***						
**Diagnosis-specific specialized outpatient care in 2001**–**05** (ref. no diagnosis-specific outpatient care)						
Somatic diagnosis	872	2.5	1.77 (1.50–2.08)	164	0.5	1.25 (0.90–1.74)
Mental diagnosis	571	4.4	3.16 (2.80–3.57)	103	0.8	2.49 (1.90–3.26)
**Diagnosis-specific inpatient care in 2001**–**05** (ref. no diagnosis-specific inpatient care)						
Somatic diagnosis	613	3.9	2.9 (2.56–3.28)	107	0.7	2.10 (1.60–2.75)
Mental diagnosis	507	10	8.26 (7.32–9.32)	90	1.8	6.31 (4.80–8.29)
Suicide attempt	314	22.0	15.86 (13.9–18.1)	46	3.2	9.20 (6.63–12.76)
**Prescribed medication dispensed in 2005** (ref. no medication)						
Antidepressants only	299	2.2	2.35 (1.97–2.79)	47	0.3	1.46 (0.99–2.15)
Anxiolytics only	109	3.2	3.40 (2.70–4.29)	23	0.7	2.83 (1.74–4.61)
Both prescribed	423	5.8	6.32 (5.37–7.45)	86	1.2	4.99 (3.55–7.02)

1 Type of living area: Big cities: Stockholm, Gothenburg and Malmo; Medium-sized cities: cities with more than 90 000 inhabitants within 30 km distance from the centre of the city; small cities/villages;

2 Single means living without partner and includes divorces, separated or widowed

Table 2 shows univariate HRs for suicide attempt and suicide with regard to the various covariates. Female sex was associated with a higher risk for suicide attempt (HR 1.2; 95% CI: 1.05–1.37) and lower risk for suicide completion (HR 0.47; 95% CI: 0.36–0.62) compared to male. With younger age (range of HRs 2.15 to 7.58) and lower education (range HRs 2.01 to 2.39), the risk for suicide attempt was higher.

Furthermore, being born outside EU and Nordic countries and being married/cohabiting without children living at home were found to be associated with a lower risk of attempted suicide (table 2).

On the other hand, individuals living without partner and with no children living at home comprised a risk group for both suicide attempt (HR 1.56; 95% CI: 1.33–1.83) and suicide (HR 2.35; 95% CI: 1.58–3.50). A history of previous health care use (in- or outpatient) due to mental diagnoses as well as suicide attempt and prescribed medication were strong predictors for suicidal behaviour (range of HRs 2.35 to 15.86) compared to those who were not treated in in- or outpatient care during 2001–05 for mental diagnoses or suicide attempt, or received antidepressants or anxiolytics during 2005.

Table 3 reports multivariate HRs and 95% CI for suicide attempt with respect to the various risk factors, where they were mutually adjusted. The results show that female sex, young age, low education, and living without partner and with no children living at home were associated with higher risk for suicide attempt (range of HRs 1.15 to 2.11) compared to male sex, age over 55 years, high education, and living with a partner and children. Additionally, previous in- and outpatient health care due to mental diagnoses (range of HRs 1.30 to 2.88), previous inpatient care due to suicide attempt (HR 3.89; 95% CI: 3.29–4.60) and prescribed medication in 2005, especially antidepressants and anxiolytics in combination (HR 3.35; 95% CI: 2.83–3.98) were strongly associated with a higher risk for suicide attempt compared to those without similar health care use and medication (Table 3).

**Table 3:** Multivariate hazard ratios with 95% confidence intervals (CI) for suicide attempt (2006–10), 46 745 women and men, aged 18–64 years and living in Sweden on 31-Dec-2004, and in 2005 on disability pension due to common mental disorders^3^.

Characteristics	Hazard ratio (95% CI)
***Socio-demographic factors***	
**Sex**	
Male	1
Female	1.15 (1.01–1.32)
**Age (years) in 2005**	
18–24	2.11 (1.55–2.89)
25–34	2.06 (1.64–2.59)
35–44	2.06 (1.69–2.52)
45–54	1.52 (1.25–1.85)
55–64	1
**Educational level (years)**	
Compulsory (0–9)	1.57 (1.29–1.91)
High school (10–12)	1.38 (1.15–1.65)
University (13 or >)	1
Unknown	0.86 (0.40–1.85)
**Family type**	
Marr/cohabit. with no children at home	0.73 (0.56–0.95)
Marr/cohabit. with children at home	1
Single^2^ no children at home	1.23 (1.04–1.45)
Single^2^ with children at home	1.29 (1.06–1.57)
***Health care factors***	
**Diagnosis-specific specialized outpatient care in 2001**–**05 (ref. no diagnosis-specific outpt. care**)	
Somatic diagnosis	1.12 (0.94–1.33)
Mental diagnosis	1.30 (1.14–1.48)
**Diagnosis-specific inpatient care in 2001**–**05 (ref. no diagnosis-specific inpatient care)**	
Somatic diagnosis	1.45 (1.26–1.67)
Mental diagnosis	2.88 (2.47–3.35)
Suicide attempt	3.89 (3.29–4.60)
**Prescribed medication dispensed in 2005 (ref. no medication)**	
Antidepressants only	1.82 (1.52–2.17)
Anxiolytics only	2.24 (1.77–2.82)
Both prescribed	3.35 (2.83–3.98)

2 Single means living without partner and includes divorces, separated or widowed.

3 all variables were mutually adjusted.

**Figure 1:**
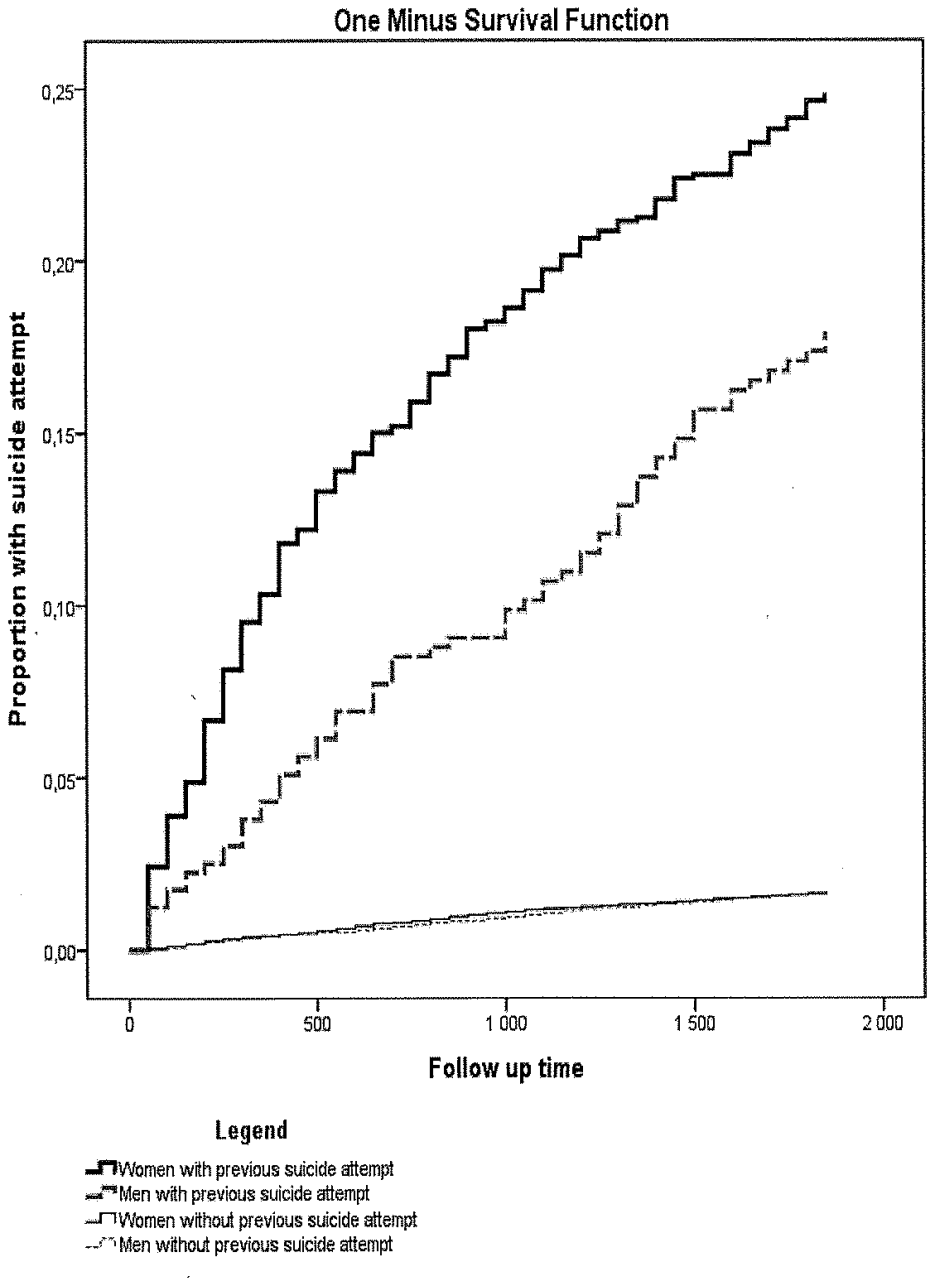
Proportion of suicide attempt, Life table estimates, 1-survival; during follow-up according to previous suicide attempt and sex.

**Figure 2:**
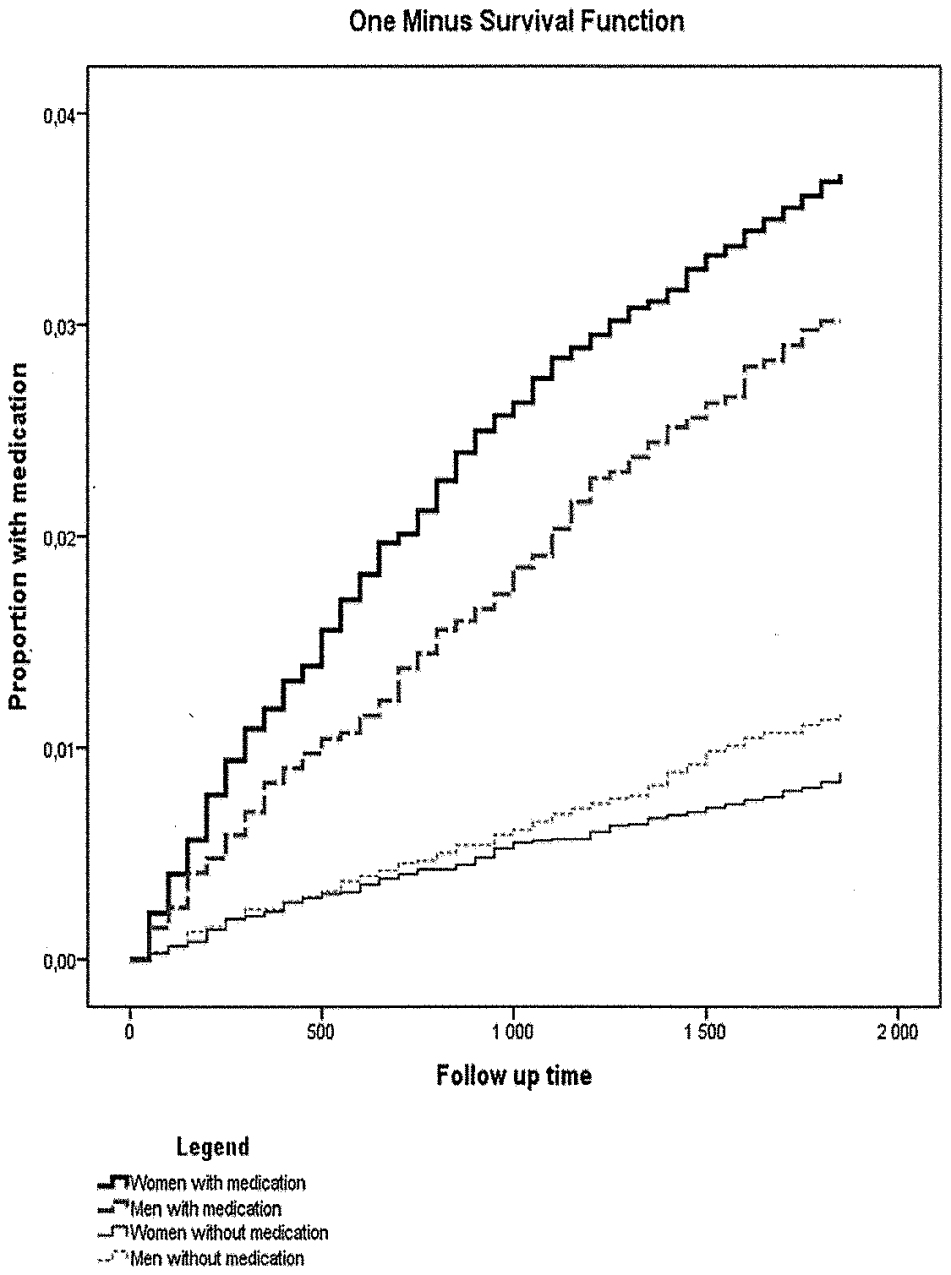
Proportion of suicide attempt, Life table estimates, 1-survival; during follow-up according to medication and sex.

Statistically significant (figure 1 and 2) interactions were observed between sex and inpatient care due to previous suicide attempt in 2001–05 (p = 0.009) and prescribed medication (p = 0.004) in 2005; no other interactions were significant. Women with previous inpatient care for suicide attempt had a higher risk for reattempting suicide (HR 4.43; 95% CI: 3.63–5.41) compared to women without inpatient care due to suicide attempt. The comparable HR for men was 2.75; 95% CI: 1.99–3.79. With regard to prescription of antidepressants and anxiolytics together, the HR for attempting suicide for women was 3.96; 95% CI: 3.19–4.92 and for men 2.48; 95% CI: 1.86–3.31 compared to medication-free individuals.

Table 4 reports multivariate HRs and 95% CI for suicide with respect to the various risk factors, where they were mutually adjusted. In this adjusted model, being male and living without partner and with no children living at home were associated with a higher risk for suicide (range of HRs 1.68 to 2.14) compared to females and living with partner and children at home. Previous inpatient care due to mental diagnoses or suicide attempt and medication, namely anxiolytics alone or in combination with antidepressants, were still strongly associated with suicide completion (range of HRs 2.1 to 3.3) compared to individuals without such inpatient care or medication.

**Table 4:** Multivariate hazard ratios with 95% confidence intervals (CI) for suicide (2006–10), 46 745 women and men, aged 18–64 years and living in Sweden on 31-Dec-2004, and in 2005 on disability pension due to common mental disorders^3^.

Characteristics	Hazard ratio (95% CI)
***Socio-demographic factors***	
**Sex**	
Male	2.14 (1.61–2.85)
Female	1
**Age (years) in 2005**	
18–24	1.23(0.59–2.60)
25–34	1.32(0.82–2.14)
35–44	0.90(0.58–1.39)
45–54	1.26(0.88–1.81)
55–64	1
**Educational level (years)**	
Compulsory (0–9)	0.90(0.62–1.32)
High school (10–12)	0.80(0.57–1.12)
University (13 or >)	1
Unknown	0.80(0.19–3.33)
**Family status**	
Marr/cohabit. with no children at home	1.23 (0.72–2.10)
Marr/cohabit. with children at home	1
Single^2^ no children at home	1.68 (1.11–2.53)
Single^2^ with children at home	1.48 (0.86–2.54)
***Health care factors***	
**Diagnosis-specific specialized outpatient care in 2001–05** (ref. no diagnosis-specific outpt. care)	
Somatic diagnosis	0.95 (0.67–1.35)
Mental diagnosis	1.23 (0.91–1.66)
**Diagnosis-specific inpatient care in 2001**–**05** (ref. no diagnosis-specific inpatient care)	
Somatic diagnosis	1.29 (0.94–1.76)
Mental diagnosis	2.71 (1.94–3.80)
Previous suicide attempt	3.00 (2.00–4.50)
**Prescribed medication dispensed in 2005** (ref. no medication)	
Antidepressants only	1.32 (0.89–1.96)
Anxiolytics only	2.10 (1.28–3.44)
Both prescribed	3.30 (2.31–4.72)

2 Single means living without partner and includes divorces, separated or widowed.

3 all variables were mutually adjusted as well as adjusted for significant confounders like age, education level, outpatient care in 2001–05.

## Discussion

Young age, low educational level, living without a partner, previous specialised health care due to mental or somatic diagnoses, previous suicide attempt, as well as prescribed psychotropic medication (both antidepressants and anxiolytics alone or combined) were associated with a higher risk of subsequent suicide attempt in individuals on disability pension due to CMD. Whereas, living without partner and no children living at home, previous inpatient care due to mental diagnoses, and previous suicide attempt along with prescribed psychiatric medication were predictive of completed suicide.

To the best of our knowledge, this is the first study ever to investigate risk factors for suicidal behaviour in individuals on disability pension due to CMD. Main strengths of the study are that it is population based, has a prospective cohort design, and is based on high quality Swedish registers [40], [41]. We could include the whole population of working age of an entire country and avoid selection and recall bias, did not have dropouts or loss to follow up, and had enough statistical power even with regard to such infrequent outcomes like suicide attempt and suicide. This study had also the opportunity to include a wide range of potential risk factors derived from different registers, including factors on socio-demographics, healthcare, previous suicide attempt, and prescribed medication.

Also limitations of this study should be mentioned. The validity of disability pension diagnoses is often discussed, however, hardly studied at all. Nevertheless, Ljungdahl *et al., in* 1991 conducted a study showing high validity of sick-leave diagnoses compared to diagnoses from medical records. Granting of disability pension is preceded by a long process of medical assessments as disability pension benefits are often paid for several years [42]. Moreover, considering the stigma around mental diagnoses, we believe that the validity of mental disability pension diagnoses is good, that is, people with a mental DP diagnoses are also likely to have such a diagnoses [43], [44]. However, this means that some disability pensioners with mental disorders were not included, as they might not have been given a mental DP diagnosis. Others might have been given a mental DP diagnosis as secondary diagnosis following a somatic main diagnoses, and thus not included in the cohort. This can be seen as both a strength and a limitation – the strength of this is that our cohort of CMD is stricter defined then when including also secondary diagnoses, the limitation is that we do not know what the effect of including them might have been for the results. Further studies need to be carried out related to these issues. Also, we used a very broad group of mental disorder rather than focusing on specific disorders. It should be mentioned, that we have considered only mental diagnoses and suicide attempts from in- and specialised outpatient care, we may have captured only the cases of greater medical severity [45] – which can be seen as both a strength and a limitation of the study.

We found that in individuals with disability pension due to CMD, female sex was associated with a higher risk for suicide attempt and male sex for suicide. To the best of our knowledge, to date there is no study investigating sex differences in people on disability pension due to CMD with regard to suicidal behaviour. Our findings are in line with studies of the general population, providing well-established results on sex differences regarding suicidal behaviour [19]–[21], [23]. Moreover, young age and low education were associated with a higher risk of suicide attempt, also in line with previous research [12], [20], [22], [46].

Our results show that living without partner and with no children living at home was associated with higher risk for suicidal behaviour among people on disability pension due to CMD. Similar results have been found previously with regard to other study populations, e.g. the general population or individuals on disability pension due to any diagnosis [20], [46]. Additionally, we found that living with partner and with no children living at home seems to have a protective influence with regard to suicide attempt compared to those who are living with partner and with children living at home. These risk and protective indicators should be taken into consideration when monitoring mental health of disability pensioners due to CMD.

We found an association between inpatient care due to somatic diagnoses and future suicide attempt. Previous research suggests that somatic disorders, for instance, cancer, stroke, epilepsy, multiple sclerosis, and different other neurological disorders are associated with an increased risk of suicidal behaviour [20], [47]. Such associations might be due to chronic pain or the terminal nature of the disease. Our observed association was independent from the effect of mental disorders in in- and specialised outpatient care and prescribed medication. Still, these measures might not entirely cover underlying mental disorders and residual confounding is likely.

Previous and ongoing in- and outpatient care due to mental diagnoses and suicide attempt turned out to be the strongest predictors for subsequent suicidal behaviour. This is in line with previously conducted studies on suicidal behaviour [12], [13], [48], [49]. Our results show that in this cohort of people on disability pension due to CMD, those with anxiolytics, either solitary or in combination with antidepressants, had a higher risk of suicidal behaviour. Moreover, statistically significant interactions were found between sex and having been prescribed psychotropic medication with regard to subsequent risk of suicide attempt. Antidepressants are recommended for treatment of depression and anxiety disorders [6], [50], [51]. Additional prescription of anxiolytics, particularly benzodiazepines is recommended for acute conditions or for individuals with predominant sleep disorders [6], [50], [51]. It has previously been reported that comorbid anxiety may worsen the prognosis and may pose an increased risk for suicidal behaviour in patients with depressive disorder [17], [52]–[54]. Additionally, association of benzodiazepines with increased suicidal behaviour have been previously reported [55]–[57], indicating that comorbid anxiety with depression treated with antidepressants in combination with benzodiazepines may contribute to an elevated risk for suicidal behaviour. Future studies are warranted in order to scrutinize the association of type, frequency, and dosage of prescribed medications and subsequent suicidal behaviour in disability pensioners due to CMD.

## Conclusion

Among the risk markers for suicidal behaviour in individuals on disability pension due to common mental disorders, along with socio-demographic factors (e.g. young age, low education, and living alone), previous health care, particularly inpatient care due to mental diagnoses or suicide attempt, and combined antidepressant and anxiolytic medication are of high importance. Further research is warranted to obtain better knowledge on risk factors for suicidal behaviour in this group at high risk of suicidal behaviour. These studies should consider specific groups of people (age, type of work, education, birth country etc.), specific diagnoses, as well as regarding different grades of disability pension.
